# Supplementary material for: Expression of a human cDNA in moss results in spliced mRNAs and fragmentary protein isoforms
Source: Commun Biol. 2021 Aug 12;4:964. doi: 10.1038/s42003-021-02486-3 (PMC8361020; doi:10.1038/s42003-021-02486-3)
Supplement: Supplementary file 1 — Supplementary Information [file 42003_2021_2486_MOESM1_ESM.pdf]

SUPPLEMENTARY INFORMATION

Title: Expression of a human cDNA in moss results in spliced mRNAs and fragmentary protein isoforms

Authors: Oguz Top, Stella W. L. Milferstaedt, Nico van Gessel, Sebastian N. W. Hoernstein, Bugra Özdemir, Eva L. Decker, Ralf Reski

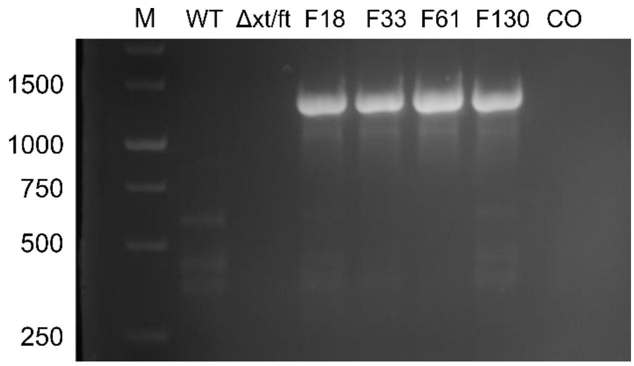

Supplementary Fig. 1: The presence of complete FIX CDS in transgenic lines. Genomic DNA from protonema cultures of 4 FIX-transgenic lines (F), the parental line Δ*xt/ft*, and WT was prepared and used for PCR using the primers FIXfwdB and FIXrevB. M: 1 kb Marker (Thermo Fisher Scientific), CO: Water control.

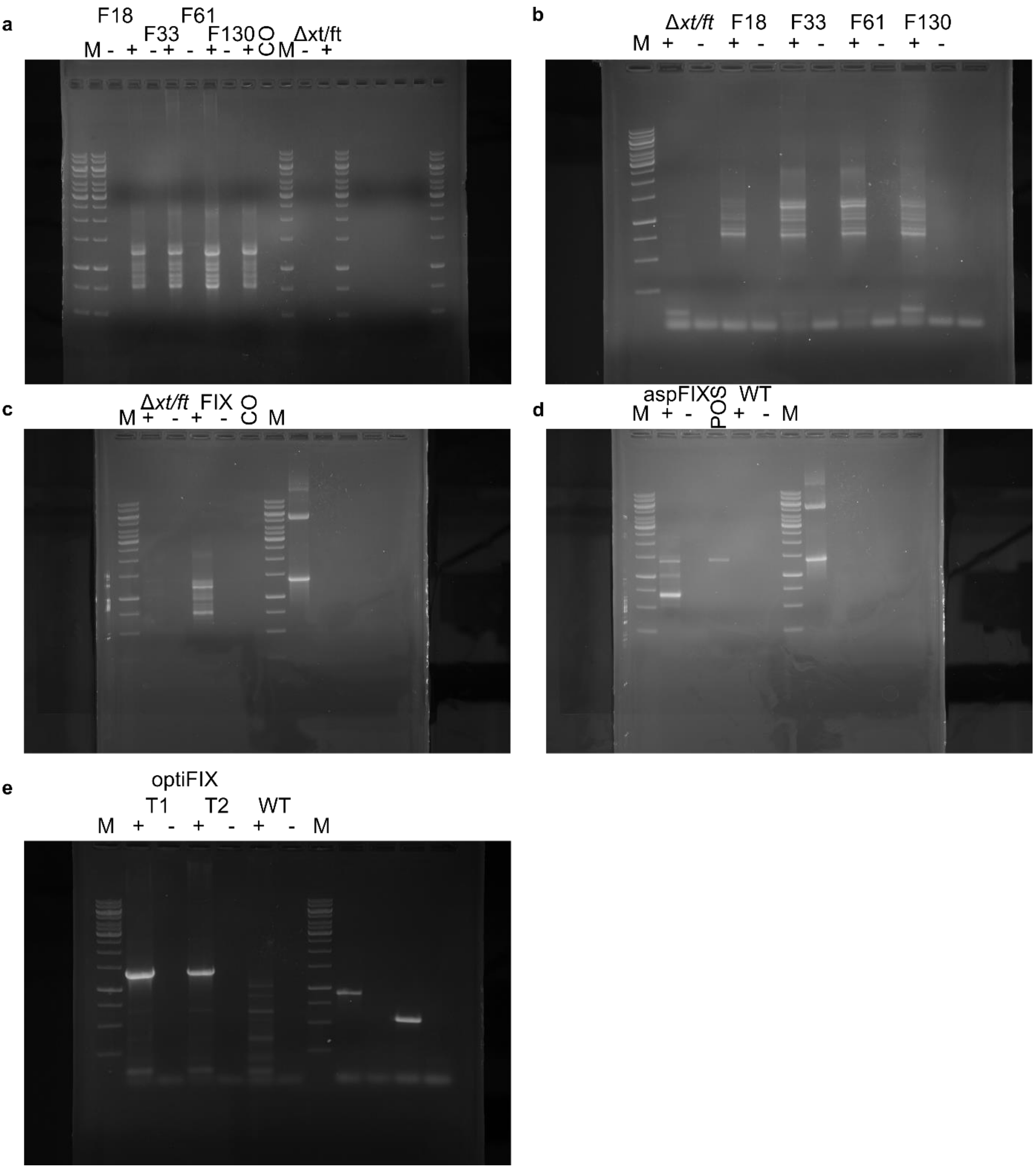

Supplementary Fig. 2: Unedited agarose gel electrophoresis images of Fig. 1a (a), Fig. 1b (b), Fig. 1c (c), Fig. 5a (d) and Fig. 5d (e). M: 1 kb Marker (Thermo Fisher Scientific), -: without reverse transcription, +: with reverse transcription, CO: Water control, Pos: PCR with aspFIX plasmid used as positive control.

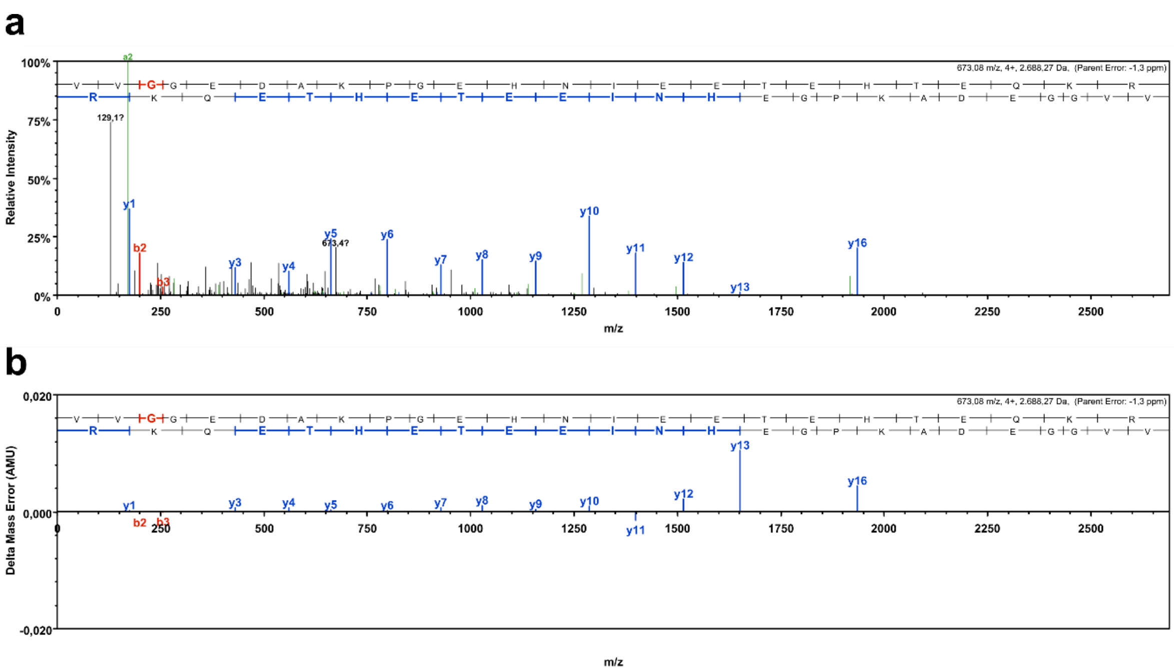

Supplementary Fig. 3: Identification of the peptide VVGGEDAKPGEHNIEETEHTEQKR by MS. a HCD fragment ion spectrum of the identified peptide. **b** Fragment mass error distribution of the b- and y-ion series.

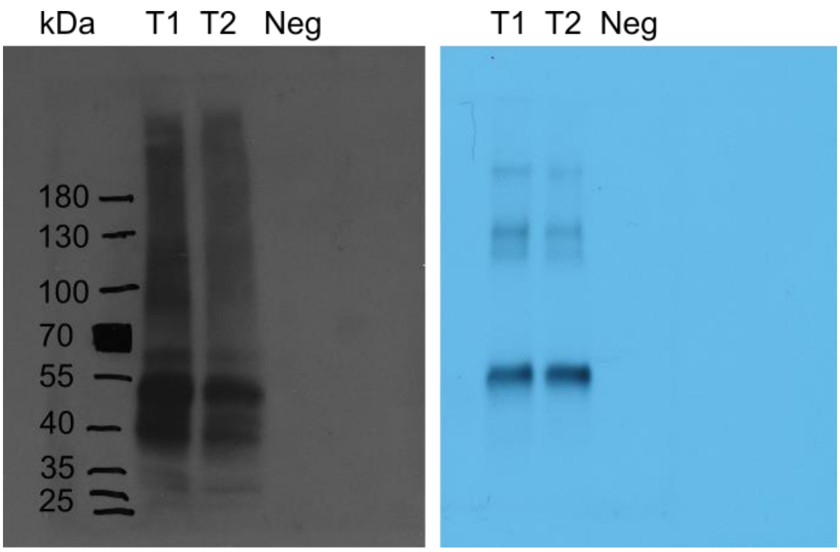

Supplementary Fig. 4: Unedited blots for Fig. 4 (Left) and Fig. 5 (Right). Immunodetection of extracellular FIX produced by two transfections (anti-FIX Ab 1:5,000). Culture supernatant of non-transformed cells was used as negative control (Neg). T1, T2: Respective Two different transient transfections with the FIX cDNA-based expression plasmid (Left) and optiFIX expression plasmid (Right). PageRuler Prestained Protein Ladder (Thermo Fisher Scientific, Catalog Number: 26616) was used and marker bands were marked on the film before scanning.

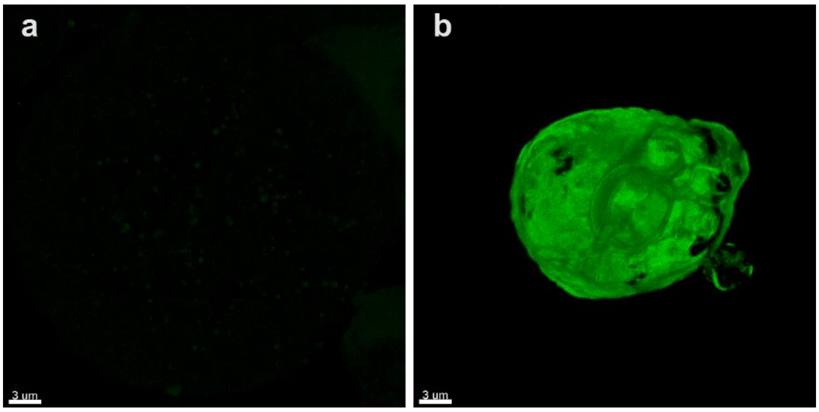

Supplementary Fig. 5: 3D rendered confocal Z-stacks of Physcomitrella protoplasts transfected with FH-Citrine (a) and optiFH-Citrine (b). Scale bar is 3 µm.

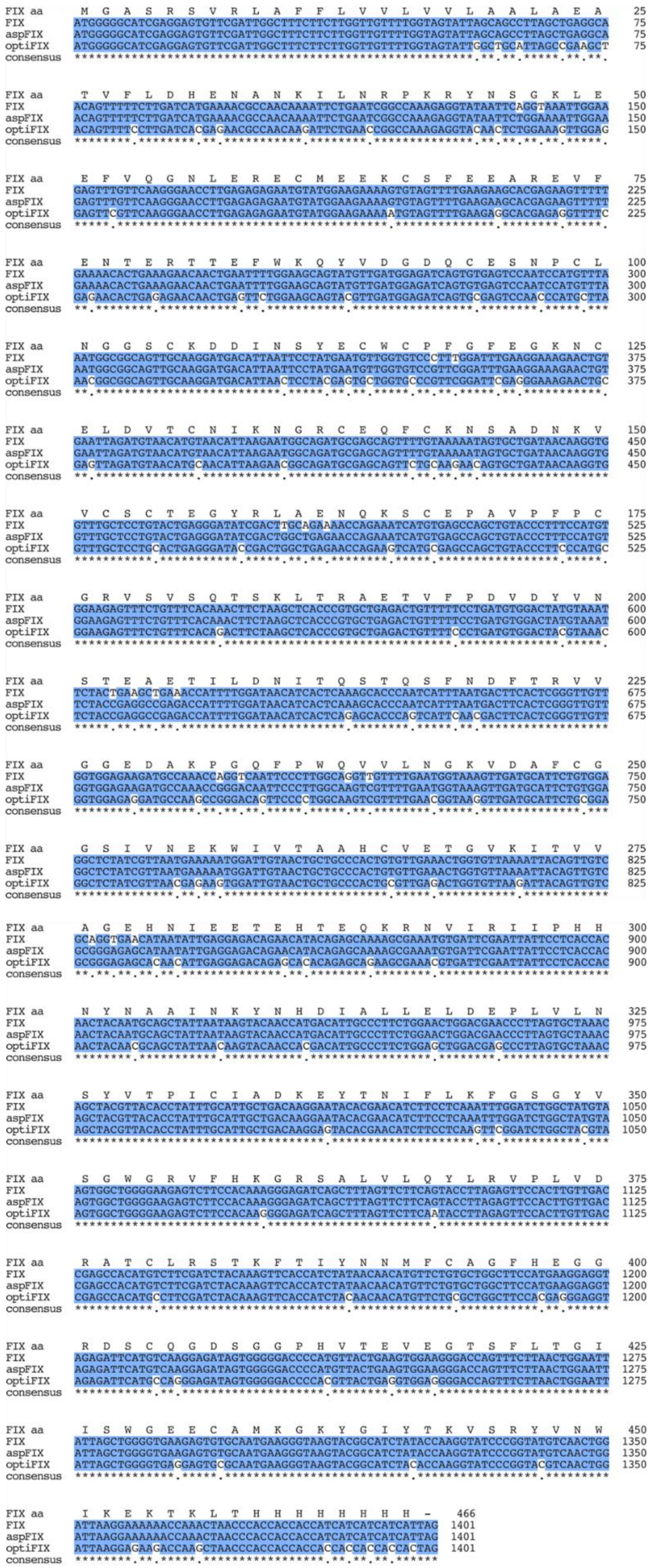

Supplementary Fig. 6: Multiple sequence alignment of FIX, aspFIX and optiFIX CDS. The translated amino acid sequence of FIX (FIX aa) was added for better visualization. Alignment was performed and formatted for publication with MUSCLE via the "MSA" package for R.

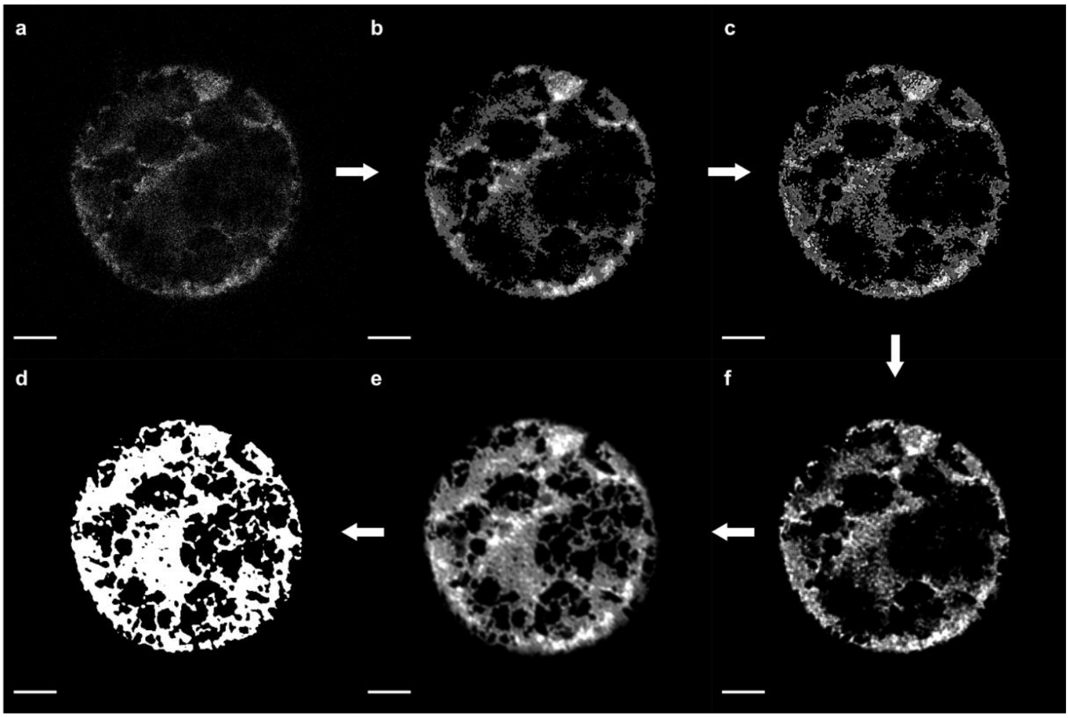

Supplementary Fig. 7: The workflow of the image processing steps demonstrated with an exemplary single slice from a FIX-Citrine cell. The original, raw image from the microscope (**a**) was first processed with a median filter (**b**). The resulting image was subjected to an unsharp-mask operation (**c**). Afterwards, a Richardson-Lucy restoration algorithm was implemented (**f**). The pre-processing was completed with a local intensity equalization operation (**e**). The final version of the image was then thresholded using a local adaptive Otsu thresholding method, leading to the binary masks (**d**) that were subsequently used for selection of the voxels for quantification. Scale bar is 5 µm.

Supplementary Table 1: Moss microRNAs targeting human FIX CDS. Analysis was performed using psRNAtarget (http://plantgrn.noble.org/v1_psRNATarget/). The settings of this analysis: Maximum expectation: 5; Length for complementarity scoring (hspsize): 20; # of top target genes for each small RNA: 200; Target accessibility - allowed maximum energy to unpair the target site (UPE): 25; Flanking length around target site for target accessibility analysis: 17 bp in upstream / 13 bp in downstream; Range of central mismatch leading to translational inhibition: 9 – 11 nt. The microRNAs as well as their binding sites, expectation score, maximum energy to unpair the target site (UPE) and mode of action of microRNAs are shown.

MicroRNA	Alignment	Expectation	UPE	Mode of action
**miR1044-3P**	`miRNA    20  UUUUGUUUAUACGUGAUGUU  1`<br>`              ::..:::::::: : ::.:`<br>`Target 1304  AAGGCAAAUAUGGAAUAUAU  1323`	5	13.996	Cleavage
**miR1028c-3P**	`miRNA   21  CGAGA-AUUUGGAUGUUACGGU  1`<br>`            :. :: :-:::: .::.:::::`<br>`Target 495  GUCCUGUGAACCAGCAGUGCCA  516`	5	21.847	Translational inhibition

Supplementary Table 2: The donor splice sites for all 87,533 annotated transcripts corresponding to the 32,926 protein-encoding genes of the current *P. patens* genome release v3.3.

Motif	*Count*	*Percentage (%)*	*Motif*	*Count*	*Percentage (%)*	*Motif*	*Count*	*Percentage (%)*
CAGGT	113,285	22.5460	*TAAGT*	1,610	0.3204	*AGAGC*	44	0.0088
AAGGT	84,769	16.8708	*AGTGT*	1,530	0.3045	*GCTGC*	44	0.0088
GAGGT	57,630	11.4695	*TGAGT*	1,492	0.2969	*ATCAT*	41	0.0082
CTGGT	20,698	4.1193	*AAGGC*	1,487	0.2959	*CGGGC*	41	0.0082
ATGGT	18,574	3.6966	*CTCGT*	1,385	0.2756	*GATGC*	41	0.0082
TTGGT	15,064	2.9980	*GGAGT*	1,330	0.2647	*TTTGC*	40	0.0080
TAGGT	12,835	2.5544	*ATCGT*	1,273	0.2534	*TCGGC*	39	0.0078
CAAGT	12,206	2.4292	*GACGT*	1,273	0.2534	*GCGGC*	36	0.0072
GTGGT	9,443	1.8793	*GTTGT*	1,259	0.2506	*GGGGC*	36	0.0072
ACGGT	9,246	1.8401	*TTCGT*	985	0.1960	*GGAGC*	35	0.0070
TGGGT	9,034	1.7980	*GAGGC*	981	0.1952	*CATGC*	34	0.0068
AGGGT	8,314	1.6547	*ACCGT*	914	0.1819	*TCAGC*	33	0.0066
TCGGT	7,588	1.5102	*TACGT*	908	0.1807	*CCGGC*	32	0.0064
CGGGT	6,757	1.3448	*CTAGT*	879	0.1749	*TTCGC*	32	0.0064
AAAGT	6,677	1.3289	*CGTGT*	870	0.1731	*ATTGC*	31	0.0062
CCGGT	6,223	1.2385	*ATAGT*	856	0.1704	*CTTGC*	31	0.0062
GCGGT	6,059	1.2059	*CCCGT*	804	0.1600	*GGTGC*	30	0.0060
AATGT	5,632	1.1209	*TCCGT*	742	0.1477	*TCTGC*	30	0.0060
CATGT	5,495	1.0936	*TGCGT*	673	0.1339	*AGTGC*	28	0.0056
GGGGT	5,371	1.0689	*AGCGT*	665	0.1323	*TGTGC*	26	0.0052
GAAGT	4,718	0.9390	*GCCGT*	516	0.1027	*CTCGC*	23	0.0046
GATGT	4,086	0.8132	*TTAGT*	506	0.1007	*TGCGC*	23	0.0046
CTTGT	3,916	0.7794	*GTCGT*	406	0.0808	*ACTGC*	22	0.0044
ATTGT	3,638	0.7240	*CGCGT*	396	0.0788	*GAAAT*	22	0.0044
AGAGT	2,987	0.5945	*GGTGT*	336	0.0669	*CCTGC*	21	0.0042
CCTGT	2,979	0.5929	*GTAGT*	300	0.0597	*GGCGC*	19	0.0038
ACTGT	2,904	0.5780	*GGCGT*	244	0.0486	*TACGC*	19	0.0038
CAGGC	2,860	0.5692	*CTGGC*	221	0.0440	*ACAGC*	18	0.0036
GCTGT	2,830	0.5632	*ATGGC*	199	0.0396	*GTTGC*	18	0.0036
TCTGT	2,772	0.5517	*TAGGC*	181	0.0360	*ATCGC*	17	0.0034
TTTGT	2,553	0.5081	*TTGGC*	89	0.0177	*TTAGC*	17	0.0034
ACAGT	2,426	0.4828	*CAAGC*	65	0.0129	*TGAGC*	16	0.0032
CCAGT	2,286	0.4550	*AAAGC*	55	0.0109	*AACGC*	15	0.0030
CACGT	2,273	0.4524	*GAAGC*	55	0.0109	*CCAGC*	15	0.0030
AACGT	1,965	0.3911	*TGGGC*	54	0.0107	*GACGC*	15	0.0030
GCAGT	1,948	0.3877	*AATGC*	52	0.0103	*GTCGC*	15	0.0030
TATGT	1,894	0.3769	*GTGGC*	52	0.0103	*GTCAT*	14	0.0028
TCAGT	1,882	0.3746	*ACGGC*	47	0.0094	*TATGC*	14	0.0028
CGAGT	1,876	0.3734	*AGGGC*	46	0.0092	*GTAGC*	13	0.0026
TGTGT	1,656	0.3296	*GCAGC*	46	0.0092	*TCCGC*	13	0.0026

Motif	*Count*	*Percentage (%)*	*Motif*	*Count*	*Percentage (%)*
AAGAT	12	0.0024	*TGCAT*	4	0.0008
AGCGC	12	0.0024	*AGAAT*	3	0.0006
ATAGC	12	0.0024	*ATGAT*	3	0.0006
CGTGC	12	0.0024	*GCTAT*	3	0.0006
CTAGC	12	0.0024	*TAATG*	3	0.0006
CGAGC	11	0.0022	*TTGAT*	3	0.0006
AAAAT	10	0.0020	*ATAAT*	2	0.0004
CAAAT	10	0.0020	*CAACT*	2	0.0004
CACGC	10	0.0020	*CCAAT*	2	0.0004
CCCGC	10	0.0020	*GACTA*	2	0.0004
GCCGC	10	0.0020	*GATAT*	2	0.0004
ACCAT	9	0.0018	*TAATA*	2	0.0004
TAAGC	9	0.0018	*TGAAA*	2	0.0004
TCAAT	9	0.0018	*TGGAT*	2	0.0004
ATTAT	8	0.0016	*CTGAT*	1	0.0002
GCAAT	8	0.0016	*CTGTG*	1	0.0002
TCTAT	8	0.0016	*CTTAT*	1	0.0002
CATAT	6	0.0012	*GGTAA*	1	0.0002
TAGAT	6	0.0012	*TAAAT*	1	0.0002
TATAT	6	0.0012	*TAACA*	1	0.0002
ACCGC	5	0.0010	*TAAGA*	1	0.0002
CAGAT	5	0.0010	*TAGAG*	1	0.0002
CGCGC	5	0.0010	*TGATG*	1	0.0002
TCGAT	5	0.0010	*TGCTT*	1	0.0002
TTCAT	5	0.0010	*TTGTC*	1	0.0002
TTTAT	5	0.0010	*TTTCG*	1	0.0002
TAAGG	4	0.0008	*TTTTA*	1	0.0002
TGAGG	4	0.0008	*TTTTT*	1	0.0002

Supplementary Table 3: The acceptor splice sites for all 87,533 annotated transcripts corresponding to the 32,926 protein-encoding genes of the current *P. patens* genome release v3.3.

Motif	*Count*	*Percentage (%)*	*Motif*	*Count*	*Percentage (%)*
CAGGT	90,834	18.0778	*AAGTG*	669	0.1331
CAGGA	54,132	10.7734	*TAGCG*	609	0.1212
CAGAT	45,474	9.0503	*AAGAG*	591	0.1176
CAGGG	36,713	7.3066	*AAGAC*	547	0.1089
CAGGC	36,350	7.2344	*AAGCA*	440	0.0876
CAGAA	22,672	4.5122	*GAGGT*	365	0.0726
CAGAG	21,613	4.3014	*AAGCC*	315	0.0627
TAGGT	19,555	3.8918	*AAGTA*	285	0.0567
CAGTT	19,239	3.8290	*AAGTC*	273	0.0543
CAGCT	18,854	3.7523	*AAGCG*	238	0.0474
CAGTG	15,897	3.1638	*GAGGA*	185	0.0368
CAGAC	14,770	2.9395	*GAGAT*	182	0.0362
CAGCA	11,526	2.2939	*GAGGG*	168	0.0334
TAGGA	9,029	1.7970	*GAGGC*	160	0.0318
CAGCC	8,266	1.6451	*GAGAA*	126	0.0251
CAGTC	7,702	1.5329	*GAGTG*	108	0.0215
TAGGG	7,378	1.4684	*GAGAG*	100	0.0199
TAGAT	7,164	1.4258	*GAGCT*	80	0.0159
CAGTA	6,745	1.3424	*GAGTT*	77	0.0153
TAGGC	6,040	1.2021	*GAGAC*	69	0.0137
CAGCG	6,004	1.1949	*GAGCA*	67	0.0133
AAGGT	4,063	0.8086	*CACAT*	51	0.0102
TAGAG	2,667	0.5308	*GAGTC*	48	0.0096
TAGAA	2,624	0.5222	*GAGTA*	46	0.0092
TAGTT	2,554	0.5083	*GAGCG*	39	0.0078
TAGTG	2,225	0.4428	*GAGCC*	37	0.0074
TAGCT	2,183	0.4345	*CACGC*	26	0.0052
TAGAC	2,119	0.4217	*CACAA*	16	0.0032
AAGGA	1,792	0.3566	*TACCT*	14	0.0028
AAGGC	1,483	0.2951	*CACAC*	11	0.0022
TAGCA	1,480	0.2946	*CACGG*	10	0.0020
AAGAT	1,342	0.2671	*TACAC*	10	0.0020
AAGGG	1,107	0.2203	*CACGT*	8	0.0016
TAGTC	967	0.1925	*TACAT*	8	0.0016
TAGCC	916	0.1823	*CACAG*	6	0.0012
AAGAA	784	0.1560	*TACAA*	6	0.0012
TAGTA	757	0.1507	*TACGA*	6	0.0012
AAGTT	685	0.1363	*TACGC*	6	0.0012
AAGCT	671	0.1335	*AACGT*	5	0.0010

Motif	*Count*	*Percentage (%)*	*Motif*	*Count*	*Percentage (%)*
AGAAT	5	0.0010	*ACGAT*	1	0.0002
CACCT	4	0.0008	*AGCAT*	1	0.0002
AACAA	3	0.0006	*ATAAT*	1	0.0002
AGGAT	3	0.0006	*CATCT*	1	0.0002
CACCA	3	0.0006	*CTAAT*	1	0.0002
CACTA	3	0.0006	*CTGGC*	1	0.0002
TACGT	3	0.0006	*GATAT*	1	0.0002
TACTC	3	0.0006	*GCTAT*	1	0.0002
AACAG	2	0.0004	*GCTTC*	1	0.0002
AACTT	2	0.0004	*GGAAG*	1	0.0002
ACTAA	2	0.0004	*GGTAT*	1	0.0002
CACTG	2	0.0004	*GTGAT*	1	0.0002
CACTT	2	0.0004	*GTTAT*	1	0.0002
GAAAT	2	0.0004	*NAGGA*	1	0.0002
GAACT	2	0.0004	*NAGGG*	1	0.0002
GACAA	2	0.0004	*NAGTG*	1	0.0002
GACAT	2	0.0004	*TACCG*	1	0.0002
GCAAT	2	0.0004	*TACTA*	1	0.0002
GCCAT	2	0.0004	*TACTG*	1	0.0002
GGCAT	2	0.0004	*TGCGT*	1	0.0002
GGGAT	2	0.0004	*TGTGA*	1	0.0002
AACAT	1	0.0002	*TTATT*	1	0.0002
ACCAT	1	0.0002	*TTTAC*	1	0.0002

Supplementary Table 4: Measured volumes and intensities in images (Supplementary Data 1) that used for Fig. 6c.

Sample	Total volume	Total intensity	Mean intensity
**FIX1**	533284	2596479	4.868848493
**FIX2**	882170	2588416	2.93414648
**FIX3**	1314016	5057256	3.848701994
**optiFIX1**	994087	27188045	27.34976416
**optiFIX2**	231369	8712866	37.65787984
**optiFIX3**	402599	28449343	70.66421675
