# Supplementary material for: Expression of a human cDNA in moss results in spliced mRNAs and fragmentary protein isoforms
Source: Commun Biol. 2021 Aug 12;4:964. doi: 10.1038/s42003-021-02486-3 (PMC8361020; doi:10.1038/s42003-021-02486-3)
Supplement: Supplementary file 2 — Description of Supplementary Files [file 42003_2021_2486_MOESM2_ESM.pdf]

Description of Additional Supplementary Files

File Name: Supplementary Data 1

Description: Zip file containing source FIX and optiFIX microscopic analysis full Z-stacks used for quantification in Fig. 6.

File Name: Supplementary Data 2

Description: Pairwise sequence alignment of full-length and predicted FH isoform based on experimentally verified transcript detected in RT-PCR.

File Name: Supplementary Data 3

Description: Pairwise sequence alignment of FH and optiFH**.**

File Name: Supplementary Data 4

Description: Amino acid sequences of full-length and predicted FH isoform (sequences used in Supplementary Data 2) based on experimentally verified transcript detected in RT-PCR without signal peptides.

File Name: Supplementary Data 5

Description: Nucleic acid sequences of FH and optiFH CDS including signal peptide (sequences used in Supplementary Data 3).

File Name: Supplementary Data 6

Description: Zip file containing source FH and optiFH microscopic analysis used in Supplementary Fig. 5.

File Name: Supplementary Data 7

Description: Nucleic acid sequences of FIX, aspFIX, and optiFIX CDS including signal peptide (sequences used in Supplementary Fig. 6).
