# Supplementary material for: Expression of a human cDNA in moss results in spliced mRNAs and fragmentary protein isoforms
Source: Commun Biol. 2021 Aug 12;4:964. doi: 10.1038/s42003-021-02486-3 (PMC8361020; doi:10.1038/s42003-021-02486-3)
Supplement: Supplementary file 4 — Supplementary Data 2 [file 42003_2021_2486_MOESM4_ESM.pdf]

Supplementary Data 2: Pairwise sequence alignment of full-length and predicted FH isoform based on experimentally verified transcript detected in RT-PCR.

```
Spliced_FH     1  EDCNELPPRRNTEILTGSWSDQTYPEGTQAIYKCRPGYRSLGNVIMVCRKGEWVALNPLR
FH             1  EDCNELPPRRNTEILTGSWSDQTYPEGTQAIYKCRPGYRSLGNVIMVCRKGEWVALNPLR
consensus      1  ************************************************************

Spliced_FH    61  KCQKRPCGHPGDTPFGTFTLTGGNVFEYGVKAVYTCNEGYQLLGEINYRECDTDGWTNDI
FH            61  KCQKRPCGHPGDTPFGTFTLTGGNVFEYGVKAVYTCNEGYQLLGEINYRECDTDGWTNDI
consensus     61  ************************************************************

Spliced_FH   121  PICEVVKCLPVTAPENGKIVSSAMEPDREYHFGQAVRFVCNSGYKIEGDEEMHCSDDGFW
FH           121  PICEVVKCLPVTAPENGKIVSSAMEPDREYHFGQAVRFVCNSGYKIEGDEEMHCSDDGFW
consensus    121  ************************************************************

Spliced_FH   181  SKEKPKCVEISCKSPDVINGSPISQKIIYKENERFQYKCNMGYEYSERGDAVCTESGWRP
FH           181  SKEKPKCVEISCKSPDVINGSPISQKIIYKENERFQYKCNMGYEYSERGDAVCTESGWRP
consensus    181  ************************************************************

Spliced_FH   241  LPSCE-------------------------------------------------------
FH           241  LPSCEEKSCDNPYIPNGDYSPLRIKHRTGDEITYQCRNGFYPATRGNTAKCTSTGWIPAP
consensus    241  *****.......................................................

Spliced_FH   246  ------------------------------------------------------------
FH           301  RCTLKPCDYPDIKHGGLYHENMRRPYFPVAVGKYYSYYCDEHFETPSGSYWDHIHCTQDG
consensus    301  ............................................................

Spliced_FH   246  ------------------------------------------------------------
FH           361  WSPAVPCLRKCYFPYLENGYNQNHGRKFVQGKSIDVACHPGYALPKAQTTVTCMENGWSP
consensus    361  ............................................................

Spliced_FH   246  ------------------------------------------------------------
FH           421  TPRCIRVKTCSKSSIDIENGFISESQYTYALKEKAKYQCKLGYVTADGETSGSIRCGKDG
consensus    421  ............................................................

Spliced_FH   246  ------------------------------------------------------------
FH           481  WSAQPTCIKSCDIPVFMNARTKNDFTWFKLNDTLDYECHDGYESNTGSTTGSIVCGYNGW
consensus    481  ............................................................

Spliced_FH   246  ------------------------------------------------------------
FH           541  SDLPICYERECELPKIDVHLVPDRKKDQYKVGEVLKFSCKPGFTIVGPNSVQCYHFGLSP
consensus    541  ............................................................

Spliced_FH   246  ------------------------------------------------------------
FH           601  DLPICKEQVQSCGPPPELLNGNVKEKTKEEYGHSEVVEYYCNPRFLMKGPNKIQCVDGEW
consensus    601  ............................................................

Spliced_FH   246  ------------------------------------------------------------
FH           661  TTLPVCIVEESTCGDIPELEHGWAQLSSPPYYYGDSVEFNCSESFTMIGHRSITCIHGVW
consensus    661  ............................................................
```

```
Spliced_FH   246 ------------------------------------------------------------
FH           721 TQLPQCVAIDKLKKCKSSNLIILEEHLKNKKEFDHNSNIRYRCRGKEGWIHTVCINGRWD
consensus    721 ............................................................

Spliced_FH   246 ------------------------------------------------------------
FH           781 PEVNCSMAQIQLCPPPPQIPNSHNMTTTLNYRDGEKVSVLCQENYLIQEGEEITCKDGRW
consensus    781 ............................................................

Spliced_FH   246 ------------------------------------------------------------
FH           841 QSIPLCVEKIPCSQPPQIEHGTINSSRSSQESYAHGTKLSYTCEGGFRISEENETTCYMG
consensus    841 ............................................................

Spliced_FH   246 ------------------------------------------------------------
FH           901 KWSSPPQCEGLPCKSPPEISHGVVAHMSDSYQYGEEVTYKCFEGFGIDGPAIAKCLGEKW
consensus    901 ............................................................

Spliced_FH   246 ------------------------------------------------------------
FH           961 SHPPSCIKTDCLSLPSFENAIPMGEKKDVYKAGEQVTYTCATYYKMDGASNVTCINSRWT
consensus    961 ............................................................

Spliced_FH   246 ------------------------------------------------------------
FH          1021 GRPTCRDTSCVNPPTVQNAYIVSRQMSKYPSGERVRYQCRSPYEMFGDEEVMCLNGNWTE
consensus   1021 ............................................................

Spliced_FH   246 -----DSTGKCGPPPPIDNGDITSFPLSVYAPASSVEYQCQNLYQLEGNKRITCRNGQWS
FH          1081 PPQCKDSTGKCGPPPPIDNGDITSFPLSVYAPASSVEYQCQNLYQLEGNKRITCRNGQWS
consensus   1081 .....*******************************************************

Spliced_FH   301 EPPKCLHPCVISREIMENYNIALRWTAKQKLYSRTGESVEFVCKRGYRLSSRSHTLRTTC
FH          1141 EPPKCLHPCVISREIMENYNIALRWTAKQKLYSRTGESVEFVCKRGYRLSSRSHTLRTTC
consensus   1141 ************************************************************

Spliced_FH   361 WDGKLEYPTCAKRHHHHHH
FH          1201 WDGKLEYPTCAKRHHHHHH
consensus   1201 *******************
```
