# Supplementary material for: Expression of a human cDNA in moss results in spliced mRNAs and fragmentary protein isoforms
Source: Commun Biol. 2021 Aug 12;4:964. doi: 10.1038/s42003-021-02486-3 (PMC8361020; doi:10.1038/s42003-021-02486-3)
Supplement: Supplementary file 5 — Supplementary Data 3 [file 42003_2021_2486_MOESM5_ESM.pdf]

Supplementary Data 3: Pairwise sequence alignment of FH and optiFH.

```
FH        ATGGCTTTCTATAAGATTTCATCTGTTTTTTTCATCTTCTGTTTCTTCTTGATTGCCCTT 60
optiFH    ATGGCTTTCTACAAGATTTCATCTGTTTTCTTCATCTTCTGCTTCTTCTTGATTGCCCTT 60
          *********** ***************** *********** ******************

FH        CCGTTCCACTCGTACGCCGAAGATTGCAATGAACTTCCTCCAAGAAGAAATACAGAAATT 120
optiFH    CCGTTCCACTCGTACGCCGAGGATTGCAACGAGCTTCCTCCAAGAAGAAACACAGAGATT 120
          ******************** ******** ** ***************** ***** ***

FH        CTGACAGGTTCCTGGTCTGACCAAACATATCCAGAAGGCACCCAGGCTATCTATAAATGC 180
optiFH    CTGACAGGTTCCTGGTCTGACCAGACATACCCAGAGGGCACCCAGGCTATCTACAAGTGC 180
          *********************** ***** ***** ***************** ** ***

FH        CGCCCTGGATATAGATCTCTTGGAAATGTAATAATGGTATGCAGGAAGGGAGAATGGGTT 240
optiFH    CGCCCTGGATACAGATCTCTTGGAAACATCATCATGGTATGCAGGAAGGGAGAGTGGGTT 240
          *********** **************  * ** ******************** ******

FH        GCTCTTAATCCATTAAGGAAATGTCAGAAAAGGCCCTGTGGACATCCTGGAGATACTCCT 300
optiFH    GCTCTTAACCCATTAAGGAAGTGCCAGAAGAGGCCCTGCGGACACCCTGGAGATACTCCT 300
          ******** *********** ** ***** ******** ***** ***************

FH        TTTGGTACTTTTACCCTTACAGGAGGAAATGTGTTTGAATATGGTGTAAAAGCTGTGTAT 360
optiFH    TTCGGTACTTTCACCCTTACAGGAGGAAACGTGTTCGAGTACGGTGTAAAGGCTGTGTAC 360
          ** ******** ***************** ***** ** ** ******** ******** 

FH        ACATGTAATGAGGGGTATCAATTGCTAGGTGAGATTAATTACCGTGAATGTGACACAGAT 420
optiFH    ACATGCAACGAGGGGTACCAGTTGCTAGGTGAGATTAACTACCGTGAGTGCGACACAGAT 420
          ***** ** ******** ** ***************** ******** ** *********

FH        GGATGGACCAATGATATTCCTATATGTGAAGTTGTGAAGTGTTTACCAGTGACAGCACCA 480
optiFH    GGATGGACCAACGATATTCCTATCTGCGAGGTTGTGAAGTGCTTACCAGTGACAGCACCA 480
          *********** *********** ** ** *********** ******************

FH        GAGAATGGAAAAATTGTCAGTAGTGCAATGGAACCAGATCGGGAATACCATTTTGGACAA 540
optiFH    GAGAACGGAAAGATTGTCAGTAGTGCAATGGAGCCAGATCGGGAGTACCACTTCGGACAG 540
          ***** ***** ******************** *********** ***** ** ***** 

FH        GCAGTACGGTTTGTATGTAACTCAGGCTACAAGATTGAAGGAGATGAAGAAATGCATTGT 600
optiFH    GCAGTACGGTTCGTATGCAACTCAGGCTACAAGATTGAGGGAGATGAGGAGATGCACTGC 600
          *********** ***** ******************** ******** ** ***** ** 

FH        TCAGACGATGGTTTTTGGAGTAAAGAGAAACCAAAGTGTGTGGAAATTTCATGCAAATCC 660
optiFH    TCAGACGATGGTTTCTGGAGTAAGGAGAAGCCAAAGTGCGTGGAGATTTCATGCAAGTCC 660
          ************** ******** ***** ******** ***** *********** ***

FH        CCAGATGTTATAAATGGATCTCCTATATCTCAGAAGATTATTTATAAGGAGAATGAACGA 720
optiFH    CCAGATGTTATCAACGGATCTCCTATCTCTCAGAAGATTATTTACAAGGAGAACGAGCGA 720
          *********** ** *********** ***************** ******** ** ***

FH        TTTCAATATAAATGTAACATGGGTTATGAATACAGTGAAAGAGGAGATGCTGTATGCACT 780
optiFH    TTCCAGTACAAGTGCAACATGGGTTACGAGTACAGTGAGAGAGGAGATGCTGTATGCACT 780
          ** ** ** ** ** *********** ** ******** *********************

FH        GAATCTGGATGGCGTCCGTTGCCTTCATGTGAAGAAAAATCATGTGATAATCCTTATATT 840
optiFH    GAGTCTGGATGGCGTCCGTTGCCTTCATGCGAGGAGAAGTCATGCGATAACCCTTACATT 840
          ** ************************** ** ** ** ***** ***** ***** ***

FH        CCAAATGGTGACTACTCACCTTTAAGGATTAAACACAGAACTGGAGATGAAATCACGTAC 900
optiFH    CCAAACGGTGACTACTCACCTTTAAGGATTAAGCACAGAACTGGAGATGAGATCACGTAC 900
          ***** ************************** ***************** *********
```

```
FH      CAGTGTAGAAATGGTTTTTATCCTGCAACCCGGGGAAATACAGCAAAATGCACAAGTACT 960
optiFH  CAGTGCAGAAACGGTTTCTACCCTGCAACCCGGGGAAACACAGCAAAGTGCACAAGTACT 960
        ***** ***** ***** ** ***************** ******** ************

FH      GGCTGGATACCTGCTCCGAGATGTACCTTGAAACCTTGTGATTATCCAGACATTAAACAT 1020
optiFH  GGCTGGATCCCTGCTCCGAGATGCACCTTGAAGCCTTGCGATTACCCAGACATTAAGCAC 1020
        ******** ************** ******** ***** ***** *********** **

FH      GGAGGTCTATATCATGAGAATATGCGTAGACCATACTTTCCAGTAGCTGTAGGAAAATAT 1080
optiFH  GGAGGTCTATACCACGAGAATATGCGACGGCCTTATTTTCCGGTAGCTGTAGGAAAGTAC 1080
        *********** ** ***********  * ** ** ***** ************** **

FH      TACTCCTATTACTGTGATGAACATTTTGAGACTCCGTCAGGAAGTTACTGGGATCACATT 1140
optiFH  TACTCCTACTACTGCGATGAGCACTTCGAGACTCCGTCAGGAAGTTACTGGGATCACATT 1140
        ******** ***** ***** ** ** *********************************

FH      CATTGCACACAAGATGGATGGTCGCCAGCAGTACCATGCCTCAGAAAATGTTATTTTCCT 1200
optiFH  CACTGCACACAGGATGGATGGTCGCCAGCAGTACCATGCCTCAGAAAGTGCTACTTCCCT 1200
        ** ******** *********************************** ** ** ** ***

FH      TATTTGGAAAATGGATATAATCAAAATCATGGAAGAAAGTTTGTACAGGGTAAATCTATA 1260
optiFH  TACTTGGAGAACGGATACAACCAGAACTACGGAAGAAAGTTCGTACAGGGTAAGTCTATC 1260
        ** ***** ** ***** ** ** **  * *********** *********** *****

FH      GACGTTGCCTGCCATCCTGGCTACGCTCTTCCAAAAGCGCAGACCACAGTTACATGTATG 1320
optiFH  GACGTTGCCTGCCACCCTGGCTACGCTCTTCCAAAGGCGCAGACCACAGTTACATGCATG 1320
        ************** ******************** ******************** ***

FH      GAGAATGGCTGGTCTCCTACTCCCAGATGCATCCGTGTCAAAACATGTTCCAAATCAAGT 1380
optiFH  GAGAACGGCTGGTCTCCTACTCCCAGATGCATCCGTGTCAAGACATGCTCCAAGTCAAGT 1380
        ***** *********************************** ***** ***** ******

FH      ATAGATATTGAGAATGGGTTTATTTCTGAATCTCAGTATACATATGCCTTAAAAGAAAAA 1440
optiFH  ATCGATATTGAGAACGGGTTCATTTCTGAGTCTCAGTACACATACGCCTTAAAGGAGAAG 1440
        ** *********** ***** ******** ******** ***** ******** ** **

FH      GCGAAATATCAATGCAAACTAGGATATGTAACAGCAGATGGTGAAACATCAGGATCAATT 1500
optiFH  GCGAAGTACCAGTGCAAGCTAGGATACGTAACAGCAGATGGTGAGACATCAGGATCAATT 1500
        ***** ** ** ***** ******** ***************** ***************

FH      ACATGTGGGAAAGATGGATGGTCAGCTCAACCCACGTGCATTAAATCTTGTGATATCCCA 1560
optiFH  ACATGCGGGAAGGATGGATGGTCAGCTCAGCCCACGTGCATTAAGTCTTGCGATATCCCA 1560
        ***** ***** ***************** ************** ***** *********

FH      GTATTTATGAATGCCAGAACTAAAAATGACTTCACATGGTTTAAGCTGAATGACACATTG 1620
optiFH  GTATTCATGAACGCCAGAACTAAGAACGACTTCACATGGTTCAAGCTGAACGACACATTG 1620
        ***** ***** *********** ** ************** ******** *********

FH      GACTATGAATGCCATGATGGTTATGAAAGCAATACTGGAAGCACCACTGGTTCCATAGTG 1680
optiFH  GACTACGAGTGCCACGATGGTTACGAGAGCAACACTGGAAGCACCACTGGTTCCATCGTG 1680
        ***** ** ***** ******** ** ***** *********************** ***

FH      TGTGGTTACAATGGTTGGTCTGATTTACCCATATGTTATGAAAGAGAATGCGAACTTCCT 1740
optiFH  TGCGGTTACAACGGTTGGTCTGATTTACCCATCTGCTACGAGAGAGAGTGCGAGCTTCCT 1740
        ** ******** ******************** ** ** ** ***** ***** ******

FH      AAAATAGATGTACACTTAGTTCCTGATCGCAAGAAAGACCAGTATAAAGTTGGAGAGGTG 1800
optiFH  AAGATCGATGTACACTTAGTTCCTGATCGCAAGAAGGACCAGTACAAGGTTGGAGAGGTG 1800
        ** ** ***************************** ******** ** ************

FH      TTGAAATTCTCCTGCAAACCAGGATTTACAATAGTTGGACCTAATTCCGTTCAGTGCTAC 1860
optiFH  TTGAAGTTCTCCTGCAAGCCAGGATTCACAATCGTTGGACCTAACTCCGTTCAGTGCTAC 1860
        ***** *********** ******** ***** *********** ***************
```

```
FH      CACTTTGGATTGTCTCCTGACCTCCCAATATGTAAAGAGCAAGTACAATCATGTGGTCCA 1920
optiFH  CACTTCGGATTGTCTCCTGACCTCCCAATCTGCAAGGAGCAGGTACAGTCATGTGGACCA 1920
        ***** *********************** ** ** ***** ***** ******** ***

FH      CCTCCTGAACTCCTCAATGGGAATGTTAAGGAAAAAACGAAAGAAGAATATGGACACAGT 1980
optiFH  CCACCAGAACTTCTCAACGGGAACGTTAAGGAGAAGACGAAGGAGGAGTACGGACACAGT 1980
        ** ** ***** ***** ***** ******** ** ***** ** ** ** *********

FH      GAAGTGGTGGAATATTATTGCAATCCTAGATTTCTAATGAAGGGACCTAATAAAATTCAA 2040
optiFH  GAGGTGGTGGAGTACTACTGCAACCCTAGATTCCTAATGAAGGGACCTAACAAGATTCAG 2040
        ** ******** ** ** ***** ******** ***************** ** *****

FH      TGTGTTGATGGAGAGTGGACAACTTTACCAGTGTGTATTGTGGAGGAGAGTACCTGTGGA 2100
optiFH  TGCGTTGATGGAGAGTGGACAACTTTACCAGTGTGCATTGTGGAGGAGAGTACCTGCGGA 2100
        ** ******************************** ******************** ***

FH      GATATACCTGAACTTGAACATGGCTGGGCCCAGCTTTCTTCCCCTCCTTATTACTATGGA 2160
optiFH  GATATCCCTGAGCTTGAGCACGGCTGGGCCCAGCTTTCTTCCCCTCCTTACTACTACGGA 2160
        ***** ***** ***** ** ***************************** ***** ***

FH      GATTCAGTGGAATTCAATTGCTCAGAATCATTTACAATGATTGGACACAGATCAATTACG 2220
optiFH  GATTCAGTGGAGTTCAACTGCTCAGAGTCATTCACAATGATTGGACACAGATCAATTACG 2220
        *********** ***** ******** ***** ***************************

FH      TGTATTCATGGAGTATGGACCCAACTTCCCCAGTGTGTGGCAATAGATAAACTTAAGAAG 2280
optiFH  TGCATTCACGGAGTATGGACCCAGCTTCCCCAGTGCGTGGCAATCGATAAGCTTAAGAAG 2280
        ** ***** ************** *********** ******** ***** *********

FH      TGCAAATCATCAAATTTAATTATACTTGAGGAACATTTAAAAAACAAGAAGGAATTCGAT 2340
optiFH  TGCAAGTCATCAAACTTAATTATCCTTGAGGAGCACTTAAAGAACAAGAAGGAGTTCGAT 2340
        ***** ******** ******** ******** ** ***** *********** ******

FH      CATAATTCTAACATAAGGTACAGATGTAGAGGAAAAGAAGGATGGATACACACAGTCTGC 2400
optiFH  CACAACTCTAACATTAGATATCGGTGTCGTGGAAAGGAGGGATGGATCCACACAGTCTGC 2400
        ** ** ******** ** **  * *** * ***** ** ******** ************

FH      ATAAATGGAAGATGGGATCCAGAAGTGAACTGCTCAATGGCACAAATACAATTATGCCCA 2460
optiFH  ATCAACGGAAGATGGGATCCAGAGGTGAACTGCTCAATGGCACAGATCCAGTTATGCCCA 2460
        ** ** ***************** ******************** ** ** *********

FH      CCTCCACCTCAGATTCCCAATTCTCACAATATGACAACCACACTGAATTATCGGGATGGA 2520
optiFH  CCTCCACCTCAGATTCCCAACTCTCACAACATGACAACCACACTGAACTATCGAGACGGT 2520
        ******************** ******** ***************** ***** ** **

FH      GAAAAAGTATCTGTTCTTTGCCAAGAAAATTATCTAATTCAGGAAGGAGAAGAAATTACA 2580
optiFH  GAAAAAGTTTCAGTTCTTTGCCAGGAGAACTACCTAATTCAGGAGGGAGAGGAGATTACA 2580
        ******** ** *********** ** ** ** *********** ***** ** ******

FH      TGCAAAGATGGAAGATGGCAGTCAATACCACTCTGTGTTGAAAAAATTCCATGTTCACAA 2640
optiFH  TGCAAGGATGGAAGATGGCAGTCAATCCCACTCTGCGTTGAGAAGATTCCATGCTCACAG 2640
        ***** ******************** ******** ***** ** ******** *****

FH      CCACCTCAGATAGAACACGGAACCATTAATTCATCCAGGTCTTCACAAGAAAGTTATGCA 2700
optiFH  CCACCTCAGATCGAGCACGGAACCATTAACTCATCCAGGTCTTCACAGGAGAGTTACGCA 2700
        *********** ** ************** ***************** ** ***** ***

FH      CATGGGACTAAATTGAGTTATACTTGTGAGGGTGGTTTCAGGATATCTGAAGAAAATGAA 2760
optiFH  CACGGGACTAAGTTGAGTTACACTTGCGAGGGTGGTTTCAGGATCTCTGAGGAGAACGAG 2760
        ** ******** ******** ***** ***************** ***** ** ** **

FH      ACAACATGCTACATGGGAAAATGGAGTTCTCCACCTCAGTGTGAAGGCCTTCCTTGTAAA 2820
optiFH  ACAACATGCTACATGGGAAAGTGGAGTTCTCCACCTCAGTGCGAGGGCCTTCCTTGCAAG 2820
        ******************** ******************** ** *********** **
```

```
FH      TCTCCACCTGAGATTTCTCATGGTGTTGTAGCTCACATGTCAGACAGTTATCAGTATGGA 2880
optiFH  TCTCCACCTGAGATTTCTCACGGTGTTGTAGCTCACATGTCAGACAGTTACCAGTACGGA 2880
        ******************** ***************************** ***** ***

FH      GAAGAAGTTACGTACAAATGTTTTGAAGGTTTTGGAATTGATGGGCCTGCAATTGCAAAA 2940
optiFH  GAGGAGGTTACGTACAAGTGCTTCGAGGGTTTCGGAATTGATGGGCCTGCAATTGCAAAG 2940
        ** ** *********** ** ** ** ***** ************************** 

FH      TGCTTAGGAGAAAAATGGTCTCACCCTCCATCATGCATAAAAACAGATTGTCTCAGTTTA 3000
optiFH  TGCTTAGGAGAGAAGTGGTCTCACCCTCCATCATGCATCAAGACAGATTGCCTCAGTTTA 3000
        *********** ** *********************** ** ******** *********

FH      CCTAGCTTTGAAAATGCCATACCCATGGGAGAGAAGAAGGATGTGTATAAGGCGGGTGAG 3060
optiFH  CCTAGCTTCGAGAACGCCATCCCCATGGGAGAGAAGAAGGATGTGTACAAGGCGGGTGAG 3060
        ******** ** ** ***** ************************** ************

FH      CAAGTGACTTACACTTGTGCAACATATTACAAAATGGATGGAGCCAGTAATGTAACATGC 3120
optiFH  CAGGTGACTTACACTTGCGCAACATACTACAAGATGGATGGAGCCAGTAACGTAACATGC 3120
        ** ************** ******** ***** ***************** *********

FH      ATTAATAGCAGATGGACAGGAAGGCCAACATGCAGAGACACCTCCTGTGTGAATCCGCCC 3180
optiFH  ATTAACAGCCGATGGACTGGTCGTCCTACGTGCAGAGACACCTCCTGCGTGAACCCGCCC 3180
        ***** *** ******* **  * ** ** ***************** ***** ******

FH      ACAGTACAAAATGCTTATATAGTGTCGAGACAGATGAGTAAATATCCATCTGGTGAGAGA 3240
optiFH  ACAGTACAGAACGCTTACATCGTGTCGAGACAGATGAGTAAGTACCCATCTGGTGAGAGA 3240
        ******** ** ***** ** ******************** ** ***************

FH      GTACGTTATCAATGTAGGAGCCCTTATGAAATGTTTGGGGATGAAGAAGTGATGTGTTTA 3300
optiFH  GTACGTTACCAGTGCAGGAGCCCTTACGAGATGTTCGGGGATGAGGAGGTGATGTGCTTA 3300
        ******** ** ** *********** ** ***** ******** ** ******** ***

FH      AATGGAAACTGGACGGAACCACCTCAATGCAAAGATTCTACAGGAAAATGTGGGCCCCCT 3360
optiFH  AACGGAAACTGGACGGAGCCACCTCAGTGCAAGGACTCGACAGGAAAGTGCGGGCCCCCT 3360
        ** ************** ******** ***** ** ** ******** ** *********

FH      CCACCTATTGACAATGGGGACATTACTTCATTCCCGTTGTCAGTATATGCTCCAGCTTCA 3420
optiFH  CCACCTATTGACAACGGGGACATTACTTCATTCCCGTTGTCAGTATACGCTCCAGCTTCA 3420
        ************** ******************************** ************

FH      TCAGTTGAGTACCAATGCCAGAACTTGTATCAACTTGAGGGTAACAAGCGAATAACATGT 3480
optiFH  TCAGTTGAGTACCAGTGCCAGAACTTGTACCAGCTTGAGGGTAACAAGCGAATCACATGC 3480
        ************** ************** ** ******************** ***** 

FH      AGAAATGGACAATGGTCAGAACCACCAAAATGCTTACATCCGTGTGTAATATCCCGAGAA 3540
optiFH  AGAAACGGACAGTGGTCAGAGCCACCAAAGTGCTTACACCCGTGCGTAATCTCCCGAGAG 3540
        ***** ***** ******** ******** ******** ***** ***** ******** 

FH      ATTATGGAAAATTATAACATAGCATTAAGGTGGACAGCCAAACAGAAGCTGTATTCGAGA 3600
optiFH  ATTATGGAGAACTACAACATCGCATTAAGGTGGACAGCCAAGCAGAAGCTGTACTCGAGA 3600
        ******** ** ** ***** ******************** *********** ******

FH      ACAGGTGAATCAGTTGAATTTGTGTGTAAACGGGGATATCGTCTTTCATCACGTTCTCAC 3660
optiFH  ACAGGTGAGTCAGTTGAGTTCGTGTGCAAGCGGGGATACCGTCTTTCATCACGTTCTCAC 3660
        ******** ******** ** ***** ** ******** *********************

FH      ACATTGCGAACAACATGTTGGGATGGGAAACTGGAGTATCCAACTTGTGCAAAAAGATAA 3720
optiFH  ACATTGCGAACAACATGCTGGGATGGGAAGCTGGAGTACCCAACTTGCGCAAAGAGATAA 3720
        ***************** *********** ******** ******** ***** ******
```
