# Supplementary figures and images for: Expression of a human cDNA in moss results in spliced mRNAs and fragmentary protein isoforms
Source: Commun Biol. 2021 Aug 12;4:964. doi: 10.1038/s42003-021-02486-3 (PMC8361020; doi:10.1038/s42003-021-02486-3)

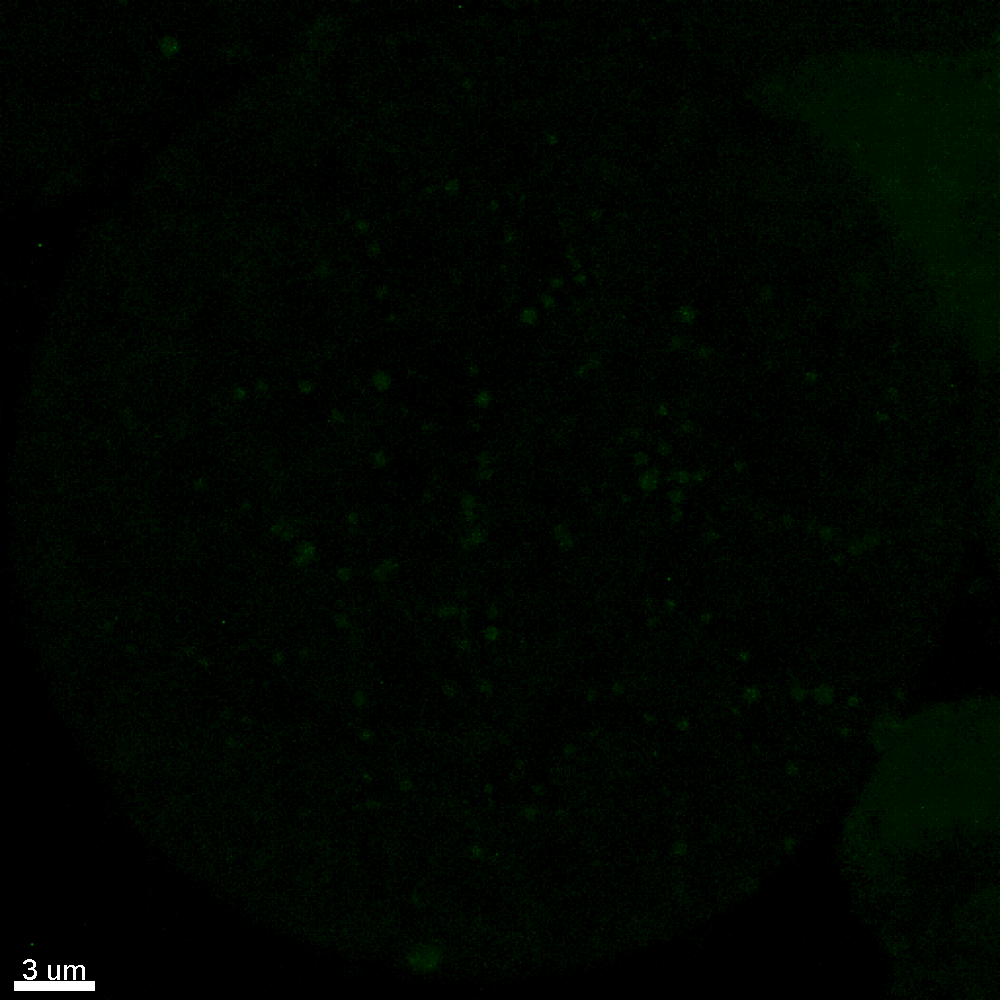

Supplement: Supplementary file 8 — Supplementary Data 6 [file 42003_2021_2486_MOESM8_ESM.zip › Supplementary Data 6/FH.tif]

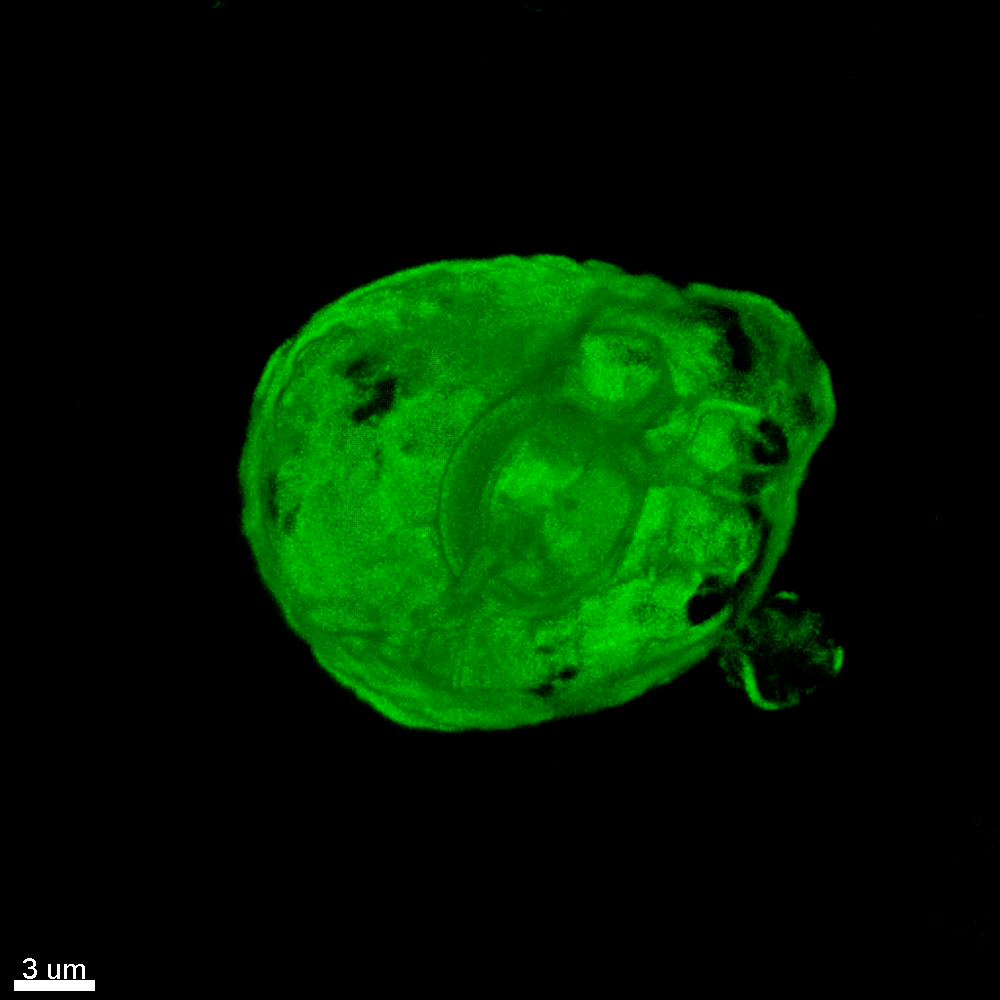

Supplement: Supplementary file 8 — Supplementary Data 6 [file 42003_2021_2486_MOESM8_ESM.zip › Supplementary Data 6/optiFH.tif]
