# Supplementary material for: Expression of a human cDNA in moss results in spliced mRNAs and fragmentary protein isoforms
Source: Commun Biol. 2021 Aug 12;4:964. doi: 10.1038/s42003-021-02486-3 (PMC8361020; doi:10.1038/s42003-021-02486-3)
Supplement: Supplementary file 10 — Reporting Summary [file 42003_2021_2486_MOESM10_ESM.pdf]

nature research

Corresponding author(s): Ralf Reski

Last updated by author(s): Jul 9, 2021

# Reporting Summary

Nature Research wishes to improve the reproducibility of the work that we publish. This form provides structure for consistency and transparency in reporting. For further information on Nature Research policies, see our Editorial Policies and the Editorial Policy Checklist.

## Statistics

For all statistical analyses, confirm that the following items are present in the figure legend, table legend, main text, or Methods section.

| n/a | Confirmed | |
|---|---|---|
| ☐ | ☒ | The exact sample size (*n*) for each experimental group/condition, given as a discrete number and unit of measurement |
| ☐ | ☒ | A statement on whether measurements were taken from distinct samples or whether the same sample was measured repeatedly |
| ☐ | ☒ | The statistical test(s) used AND whether they are one- or two-sided<br>*Only common tests should be described solely by name; describe more complex techniques in the Methods section.* |
| ☒ | ☐ | A description of all covariates tested |
| ☒ | ☐ | A description of any assumptions or corrections, such as tests of normality and adjustment for multiple comparisons |
| ☒ | ☐ | A full description of the statistical parameters including central tendency (e.g. means) or other basic estimates (e.g. regression coefficient) AND variation (e.g. standard deviation) or associated estimates of uncertainty (e.g. confidence intervals) |
| ☐ | ☒ | For null hypothesis testing, the test statistic (e.g. $F$, $t$, $r$) with confidence intervals, effect sizes, degrees of freedom and $P$ value noted<br>*Give P values as exact values whenever suitable.* |
| ☒ | ☐ | For Bayesian analysis, information on the choice of priors and Markov chain Monte Carlo settings |
| ☒ | ☐ | For hierarchical and complex designs, identification of the appropriate level for tests and full reporting of outcomes |
| ☒ | ☐ | Estimates of effect sizes (e.g. Cohen's *d*, Pearson's *r*), indicating how they were calculated |

*Our web collection on statistics for biologists contains articles on many of the points above.*

## Software and code

Policy information about availability of computer code

| | |
|---|---|
| Data collection | No custom code used to collect data. The data for microscopic and mass spectrometric analysis were collected via Leica TCS SP8 microscope (Leica Microsystems, Wetzlar, Germany) and QExactive Plus mass spectrometer (Thermo Fisher Scientific, Bremen, Germany) softwares, respectively. |
| Data analysis | All softwares used is stated in the Material and Methods and Supplementary Information.<br>Splicing motif search: R and the package ggseqlgo<br>The custom code for image processing: The code used for the image processing and quantification can be obtained from www.plant-biotech.uni-freiburg.de<br>MS analysis: Mascot Distiller V2.5.1.0 (Matrix Science, USA), Mascot V2.6.0, Scaffold4 Version 4.11.0 (Proteome Software Inc., Portland, OR) and X!Tandem<br>The custom made JavaScript-based web application, physCO, is available at www.plant-biotech.uni-freiburg.de |

For manuscripts utilizing custom algorithms or software that are central to the research but not yet described in published literature, software must be made available to editors and reviewers. We strongly encourage code deposition in a community repository (e.g. GitHub). See the Nature Research guidelines for submitting code & software for further information.

## Data

Policy information about availability of data

All manuscripts must include a data availability statement. This statement should provide the following information, where applicable:

- Accession codes, unique identifiers, or web links for publicly available datasets
- A list of figures that have associated raw data
- A description of any restrictions on data availability

All data generated or analyzed during this study are included in this article and its supplementary information. JavaScript-based web application, physCO, as well as the code used for the image processing and quantification are available at www.plant-biotech.uni-freiburg.de.

# Field-specific reporting

Please select the one below that is the best fit for your research. If you are not sure, read the appropriate sections before making your selection.

☒ Life sciences ☐ Behavioural & social sciences ☐ Ecological, evolutionary & environmental sciences

For a reference copy of the document with all sections, see nature.com/documents/nr-reporting-summary-flat.pdf

# Life sciences study design

All studies must disclose on these points even when the disclosure is negative.

| | |
|---|---|
| Sample size | Similar studies as well as previous preliminary experiments were taken into account to decide sample sizes. |
| Data exclusions | No data were excluded from the analyses. |
| Replication | All experimental findings could be reliably reproduced. |
| Randomization | Randomization is of no relevance to our work. |
| Blinding | We were not blinded to group allocation during data collection, since our target samples are transformed cells in microscopic analysis. The transformed cells were selected randomly to be analyzed. |

# Reporting for specific materials, systems and methods

We require information from authors about some types of materials, experimental systems and methods used in many studies. Here, indicate whether each material, system or method listed is relevant to your study. If you are not sure if a list item applies to your research, read the appropriate section before selecting a response.

### Materials & experimental systems

| n/a | Involved in the study | |
|---|---|---|
| ☐ | ☒ | Antibodies |
| ☒ | ☐ | Eukaryotic cell lines |
| ☒ | ☐ | Palaeontology and archaeology |
| ☒ | ☐ | Animals and other organisms |
| ☒ | ☐ | Human research participants |
| ☒ | ☐ | Clinical data |
| ☒ | ☐ | Dual use research of concern |

### Methods

| n/a | Involved in the study | |
|---|---|---|
| ☒ | ☐ | ChIP-seq |
| ☒ | ☐ | Flow cytometry |
| ☒ | ☐ | MRI-based neuroimaging |

## Antibodies

| | |
|---|---|
| Antibodies used | Primary antibody: Polyclonal anti-factor IX antibody, F0652, SIGMA ALDRICH (now Merck) - Secondary antibody: Horseradish peroxidase-linked anti-rabbit IgG, NA934, GE Healthcare |
| Validation | As it can be seen from the product website, there are ten studies which used this primary antibody. There is no report so far regarding the use of this antibody to detect moss made recombinant protein. However, our mass spectrometric analysis that can be seen in the manuscript confirmed that the signals detected by this primary antibody were correct. |
